# Molecular and Biochemical Characterization of YeeF/YezG, a Polymorphic Toxin-Immunity Protein Pair From *Bacillus subtilis*

**DOI:** 10.3389/fmicb.2020.00095

**Published:** 2020-02-14

**Authors:** Soni Kaundal, Amar Deep, Gundeep Kaur, Krishan Gopal Thakur

**Affiliations:** Structural Biology Laboratory, G. N. Ramachandran Protein Centre, Council of Scientific & Industrial Research-Institute of Microbial Technology (CSIR-IMTECH), Chandigarh, India

**Keywords:** contact-dependent antagonism, toxin-immunity system, polymorphic toxin system, bacterial toxin, protein-protein interactions

## Abstract

Polymorphic toxins are important and widespread elements of bacterial warfare that help in restricting the growth of competitors, aiding kin selection, and shaping the bacterial communities. Although widespread, polymorphic toxin systems (PTS) have been extensively studied in Gram-negative bacteria, there are limited studies describing PTS in Gram-positive bacteria. The present study characterizes YeeF/YezG, a predicted member of a PF04740 family of the polymorphic toxin-immunity system from a Gram-positive bacteria *Bacillus subtilis*. The expression of the C-terminal toxic domain of YeeF (YeeF-CT) causes growth inhibition and gross morphological changes in *Escherichia coli.* The observed toxic effects are neutralized by the co-expression of *yezG*, a gene present downstream of *yeeF*, confirming YeeF-CT/YezG as a toxin/immunity protein pair. Biochemical and *in vivo* studies reveal that YeeF-CT causes toxicity due to its non-specific metal-dependent DNase activity. This is different from the previously reported RNase activity from the three *B. subtilis* toxins belonging to PF04740 family. Isothermal titration calorimetry (ITC) data analysis suggests that YeeF-CT binds YezG with a dissociation constant in the nanomolar range. Analytical ultracentrifugation studies revealed that YeeF-CT forms a homodimer and binds with two molecules of monomeric YezG immunity protein to form a 2:2 stochiometric heterotetrameric complex. Biolayer interferometry and electrophoretic mobility shift assays show that YeeF-CT/YezG/DNA forms a stable ternary complex implicating that YezG is an exosite inhibitor of YeeF-CT. This study extends the molecular targets of the toxins in the PF04740 family and thus, this family of toxins can be broadly classified as nucleases harboring either DNases or RNases activities.

## Introduction

Though most of the microbes are uni-cellular, yet they display several complex social traits like communication, swarming motility, regulation of coordinated gene expression, etc., similar to higher multi-cellular organisms. They compete or cooperate with neighboring microbes for limited space and nutrients for survival (Visick and Fuqua, 2005; Keller and Surette, 2006). During the course of evolution, microbes have evolved several strategies to communicate, compete or cooperate with each other (Waters and Bassler, 2005; Keller and Surette, 2006; Aoki et al., 2010; Whitney et al., 2017). To restrict the growth of competitors and promote kin selection, microbes secrete several proteinaceous and non-proteinaceous toxic molecules (Zhang et al., 2011, 2012). Polymorphic toxins belong to one of these diverse and widespread family of toxins produced by bacteria that help in restricting the growth of competitors, aiding kin selection, and shaping the bacterial community (Zhang et al., 2011, 2012).

**FIGURE 1:**
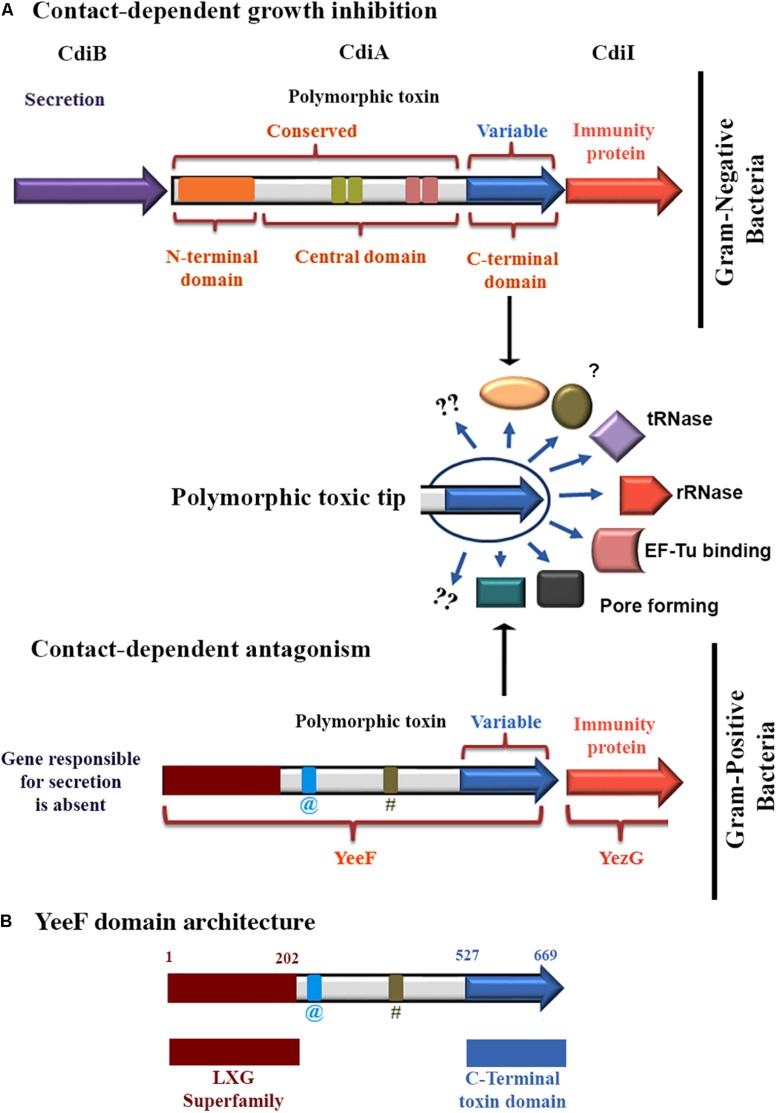
A schematic representation of the polymorphic toxin systems. **(A)** The schematic representation depicting the organization of polymorphic toxin system involved in contact-dependent growth inhibition in Gram-negative bacteria. An optional dedicated transport system may be encoded in the vicinity of toxin/immunity genes of the toxin. In a defined family, the polymorphic toxin is composed of a conserved N-terminal region having a transporter domain and a variable C-terminal region that codes for the diverse toxin modules. Apart from the transporter domain, the longer central region shows a high variation in the length and contains various combinations of domains/repeats like filamentous hemagglutinin, recombinant hot spot, etc. This central region also presumably helps in toxin display on the cell surface. Operon organization of a polymorphic toxin system involved in contact-dependent antagonism in Gram-positive bacteria. **(B)** YeeF domain architecture of YeeF toxin. The N-terminal region has a similarity with conserved LXG domain superfamily of Pfam PF04740, which is required for the secretion of toxin by type VII secretion system (Whitney et al., 2017). The C-terminal toxic module is annotated as a putative DNA/RNA non-specific endonuclease. @ Coiled-coil region; # Low complexity region. Of note that the domain architecture representation is not as per the scale.

Aravind’s group reported comprehensive bioinformatics analyses of polymorphic toxin systems (PTS) (Zhang et al., 2011, 2012) and predicted numerous polymorphic toxin and cognate immunity protein modules in both Gram-positive and Gram-negative bacteria. Polymorphic toxins are multi-domain proteins, mainly involved in inter-bacterial conflicts (Zhang et al., 2011, 2012). The product of the immunity gene neutralizes the toxin and protects the toxin-producing cells from auto-inhibition (Zhang et al., 2012). PTS have the following domain characteristics: an N-terminal trafficking domain, a central domain, and a highly variable C-terminal domain. The N-terminal trafficking domain in a toxin is conserved among related toxins belonging to the same family, and is involved in the export of the toxin to the outer membrane (Aoki et al., 2005; Zhang et al., 2011, 2012). The optional central domain varies in length and may contain repeats of several filamentous domains like hemagglutinin, recombinant hotspot, etc. The role of the central domain has not been investigated so far, but probably, this region aids in displaying the toxic module on the cell surface (Aoki et al., 2005; Zhang et al., 2011, 2012). Interestingly, the N-terminal and central regions are conserved in a PTS family, but the C-terminal domain is highly variable and relatively smaller in size harboring toxic activity (Figure 1A). The C-terminal domain displays both sequence and functional diversity with some of the annotated functions including nucleases, pore-forming toxins, nucleic acid modifying enzymes, peptidases, and protein-modifying enzymes such as ADP-ribosyl transferases (Aoki et al., 2005; Zhang et al., 2011, 2012; Jamet and Nassif, 2015). Several polymorphic toxin/immunity families have been predicted in bacteria, but only a few have been characterized experimentally. PTS family includes Contact-Dependent growth Inhibition (CDI) systems (Aoki et al., 2005, 2010), Rearrangement Hot Spot (RHS) toxins (Poole et al., 2011) and multiple adhesion family (*maf*) toxins (Jamet et al., 2015) that have been studied in Gram-negative bacteria, whereas Pfam PF04740 toxins (Holberger et al., 2012) have been studied in Gram-positive bacteria. Among all of these, CDI is the most studied phenomenon where CDI^+^ cells inhibit the growth of CDI^–^ cells by direct cell to cell contact. CDI is mediated by *cdiBAI* gene cluster, where CdiA is the toxin molecule, CdiB is a predicted outer membrane protein which facilitates the export of CdiA toxin outside the cell surface, and CdiI is the cognate immunity protein (Aoki et al., 2005, 2010; Hayes et al., 2014). Besides these essential components host factor CysK also plays an essential role in mediating CDI in *E. coli* UPEC536 (Diner et al., 2012; Kaundal et al., 2016). While CDI systems have been reported from Gram-negative bacteria, a few studies refer to the presence of similar systems in Gram-positive bacteria as contact-dependent antagonism (Koskiniemi et al., 2013; Jamet et al., 2017, 2018; Whitney et al., 2017). The first report of contact-dependent growth antagonism in Gram-positive bacteria was published by Koskiniemi et al. (2013), where WapA polymorphic toxin family of *B. subtilis* was characterized. The C-terminal domain of WapA protein harbors toxin and is involved in contact-dependent growth antagonism of susceptible strains. Whitney et al. (2017) reported that the LXG toxins (TelB and TelC), secreted by type VII secretion system of *Streptococcus intermedius*, a Gram-positive bacteria, conferred a competitive advantage against sensitive strains of *S. intermedius* and other strains of Gram-positive species (*Streptococcus pyogenes and Enterococcus faecalis*). These toxins were then neutralized by the proteins encoded by the genes immediately downstream of *telB* and *telC*. Holberger et al. (2012) have distinguished polymorphic toxin modules of Pfam PF04740 family (LXG family) from Gram-positive bacteria. These toxins show the growth inhibitory activity when expressed in *E. coli*, and co-expression of the cognate immunity protein neutralizes toxicity and prevent autoinhibition. The members of Pfam PF04740 family were characterized and it revealed the RNase activity in YobL-CT, YxiD-CT, and YqcG-CT domains from *B. subtilis* 168 (Holberger et al., 2012). Although included in the study, the C-terminal toxic domain of YeeF was not functionally characterized (Holberger et al., 2012).

In order to understand the strategies employed by bacteria to outcompete their competitors, we made attempts to characterize one of the uncharacterized toxin-immunity protein modules, a member of Pfam PF04740 polymorphic toxin family, from *B. subtilis subsp. spizizenii str. W23*. The C-terminal toxic domain from *B. subtilis* 168 shares 82% sequence identity with the annotated YeeF-CT from *Bacillus subtilis subsp. spizizenii str. W23* characterized in this study. The bioinformatics and the conserved domain database analyses suggests that the N-terminal region of YeeF belongs to a conserved Pfam PF04740, and it harbors a putative nuclease (predicted ribonuclease at UniProt KB) domain in its C-terminal region (Figures 1A,B). We performed detailed biophysical and biochemical investigations on the C-terminal toxic domain of YeeF, henceforth called YeeF-CT, a member of the PF04740 toxin system (Figures 1A,B). Here we report that YeeF-CT is a metal ion dependent DNase which is neutralized by YezG, a protein encoded by a gene downstream to *yeeF*. Hence, YeeF/YezG forms a functional toxin/immunity pair. This study suggests that members of the PF04740 family are nucleases that either have RNase or DNase activity.

## Materials and Methods

### Cloning of *yeeF-CT, yeeF-CT^*H*581*A*^*, and *yezG*

DNA region coding for *yeeF-CT* (residue range Ile527 to Gly669) was cloned in pBADMyc-His A_Modi vector (modified vector where *Nhe*I restriction site and N-terminal 6 × His tag is introduced) between *Nhe*I and *Hin*dIII restriction sites. The *yezG* gene was cloned between the restriction sites, *Nde*I/*Xho*I in pET28a vector to produce N-terminal 6× His tagged proteins. The *yeeF-CT^*H*581*A*^* henceforth called *yeeF-CT^(M)^*, a point variant was created using PCR based site-directed mutagenesis. The detailed list of constructs used in the present study is provided in the Supplementary Table S1.

### Growth Curve and CFU Counting

*Escherichia coli* BL21 (DE3) cells expressing YeeF-CT, YeeF-CT^(M)^, YeeF-CT/YezG, and pBADMyc-His A_Modi (negative control) were grown overnight at 37°C in LB media supplemented with appropriate antibiotics. Overnight grown cultures were diluted into a fresh LB medium to OD_600_ of 0.07, then grown to OD_600_ of 0.4 at 37°C, and induced with 0.1% L-arabinose. The expression of YezG was induced with 0.3 mM IPTG at 0.2 OD_600_ before the induction of YeeF-CT. The OD_600_ for each sample was measured and plotted against time. For colony counting, samples were withdrawn at 0, 1, 2, and 4-hour post-induction. The ten-fold serial dilutions i.e., 10^–3^, 10^–4^, and 10^–5^ were prepared in LB media and 10 μL of the diluted samples were plated on LB agar plates with appropriate antibiotics. The plates were incubated at 37°C overnight, and colonies were counted using a colony counter (Heathrow Scientific) and plotted against time. The experiments were done in triplicates. Both the growth curves and CFU data were plotted using Origin software 6.1, and the uncertainties were calculated using standard deviation.

### SDS-PAGE Analysis

Cells were pelleted and lysed using lysis buffer (20 mM HEPES pH 7.5, 150 mM NaCl, and a cocktail of EDTA-free protease inhibitors) by sonicating the samples for 10 min and the lysate was fractionated into supernatant and pellet. About 10 μg of total protein from supernatant fraction was separated on SDS polyacrylamide gel and detected by staining with Coomassie dye.

### Transmission Electron Microscope (TEM)

*Escherichia coli* BL21 (DE3) cells expressing YeeF-CT, YeeF-CT^(M)^, YeeF-CT/YezG and pBADMyc-His A_Modi (negative control) were analyzed by TEM. The cells were grown, as mentioned in the above section “Materials and Methods,” and were then harvested at 0, 30, 60, and 120 min post-induction. Cells were washed twice with 1 × PBS, resuspended in PBS and 30 μL of each sample was loaded on the carbon-coated copper grids 300 mesh (Polysciences, Inc.). The grids were then air-dried and loaded in the sample holder and imaged using JEM 2100 transmission electron microscope, operated at 200 keV (JEOL).

### Genomic DNA Extraction and *in vitro* DNase Activity

The cells expressing YeeF-CT, YeeF-CT^(M)^, YeeF-CT/YezG and pBADMyc-His A_Modi (negative control) were harvested at 12000g. The genomic DNA was isolated using QIAamp DNA mini kit from QIAGEN. The purified DNA samples were resolved on 0.8% agarose gel. For *in vitro* DNase activity assays, purified YeeF-CT (250 nM–2 μM) or YeeF-CT^(M)^ (500 nM–2 μM) and were incubated with the plasmid (∼180 ng) in the buffer (20 mM HEPES pH 7.5, 150 mM NaCl) at 37°C for 30 min. Then the samples were resolved on 0.8% agarose gel and visualized in UV transilluminator. To test the metal-dependent DNase activity and identify the metal ion responsible for activating the DNase activity of YeeF-CT, the toxin was chelated with 10 mM EDTA for 2 h at room temperature and dialyzed against a buffer 20 mM HEPES pH 7.5, 150 mM NaCl for 12 h. The dialyzed YeeF-CT (2 μM) was incubated with the mentioned concentration of different metal ions and tested for DNase activity, by being incubated with plasmid DNA for 30 min at 37°C. To investigate the protective role of YezG, YeeF-CT (2 μM) was pre-incubated with different concentrations of YezG (0.5–2 μM) for 45 min at room temperature and then incubated with DNA. In order to see the optimum time required for DNA cleavage by YeeF-CT, the metal-free YeeF-CT supplemented with 10 μM MnCl_2_ was incubated with plasmid DNA ranging from 1 to 60 min at 37°C. To investigate the optimum temperature for DNase activity, the metal-free YeeF-CT supplemented with 10 μM MnCl_2_ was incubated with plasmid DNA at 4, 22, 37, and, 55°C for 30 min. Similarly, YeeF-CT (2 μM) was dialyzed in buffers with different pH (4.5–9.0) levels and different NaCl concentrations (0–550 mM) for 12 h. The dialyzed protein was incubated with plasmid DNA for 30 min at 37°C. The samples in all the above experiments were resolved on 1% agarose gel and visualized under UV light.

### Confocal Microscopy Imaging

*Escherichia coli* BL21 (DE3) cells expressing YeeF-CT, YeeF-CT^(M)^, YeeF-CT/YezG, and pBADMyc-His A_Modi (negative control) were used for confocal microscopy. The cells were grown and induced as indicated in the section “Growth Curve and CFU Counting.” For confocal microscopy, the samples were washed twice in 1 × PBS and then fixed with 4% paraformaldehyde for 15 min at room temperature. Samples were washed twice in 1 × PBS, incubated with DAPI for 30 min at 37°C, then washed again with 1 × PBS twice and finally re-suspended in 1 × PBS and used for confocal microscopy. For confocal microscopy, 10 μL of each sample was loaded on the slide coated with 0.7% agarose, covered with a cover-slip, air-dried and imaged with a confocal microscope (Nikon A1R), using a 100 × oil-immersion objective and 1 Airy unit aperture. Samples were excited using an excitation wavelength of 405 nm.

### Protein Purification

*Escherichia coli* BL21 (DE3) cells expressing YeeF-CT/YeeF-CT^(M)^/YezG were plated on LB agar plates having specific antibiotics and were incubated at 37°C overnight. A single colony from the plate was inoculated in 5 mL LB media for primary culture and incubated overnight at a constant shaking of 200 rpm at 37°C. For secondary culture, 1% of primary culture was inoculated, and cells were induced by 0.1% L- arabinose/0.3 mM IPTG at 0.4–0.5 OD_600_ and further incubated at 37°C for 2 h at a constant shaking of 200 rpm. Cells were pelleted by centrifugation at 6000g for 15 min at 4°C, and the pellets were re-suspended in lysis buffer (20 mM HEPES pH 7.5, 150 mM NaCl, and a cocktail of EDTA-free protease inhibitors) followed by sonication. The supernatant was collected after centrifugation at 18000g for 45 min. All the 6× His tagged proteins were purified using Ni-NTA based affinity purification method, by following the manufacturer’s instructions. The fractions were further loaded and checked on SDS-PAGE before proceeding to the next step. The desired elution fractions were pooled and concentrated using ultrafiltration centrifugal devices (Pall Corporation). Proteins were further purified by gel filtration chromatography using Superdex 200 Increase 10/300 GL column (GE Healthcare). The identities of the protein samples were confirmed by mass spectrometry, and the purities were examined using SDS-PAGE.

### Analytical Ultracentrifugation (AUC)

To determine the oligomeric state of YeeF-CT^(M)^ and YezG and their binding stoichiometry, AUC experiments were carried out using Beckman-Coulter XL-A analytical ultracentrifuge equipped/provided with a TiAn50 eight-hole rotor. Sedimentation velocity experiments were conducted using a 2-channel epon centerpiece (12 mm) and a quartz window. Protein samples were run at three different concentrations (8, 16, and 32 μM) in buffer 20 mM HEPES, pH 7.5 and 150 mM NaCl and absorbance scans were recorded at 220, 260, and 280 nm at every 3 min interval at 40,000 rpm at 25°C. To study the binding stoichiometry of YeeF-CT^(M)^/YezG complex, purified proteins were mixed (in varying molar ratios as mentioned in section “Result”) and incubated at 4°C for 1 h before conducting analytical ultracentrifugation (AUC) experiments. The data were fit by using SEDFIT (Schuck et al., 2002) for continuous distribution c(s). SEDNTERP (Laue et al., 1992) was used to calculate the solvent density (ρ) and viscosity (η) from the chemical composition of different components of the buffer.

### Isothermal Titration Calorimetry (ITC)

To determine the K_D_, stoichiometry, and thermodynamics of YeeF-CT^(M)^/YezG binding, ITC experiments were performed using MicroCal VP-ITC (GE Healthcare) at 25°C. Both the purified proteins were dialyzed using a dialysis membrane of 3500 MWCO against the buffer (20 mM HEPES pH 7.5, 150 mM NaCl). The samples were degassed using MicroCal ThermoVac (GE Healthcare) prior to the experiments. YeeF-CT^(M)^ (10 μM; sample cell) was titrated with YezG (300 μM; syringe) with a constant stirring speed of 307 rpm. The reference power and injection volume were kept 5 μcal/sec and 6 μL, respectively. Since YeeF-CT^(M)^ is a dimer as per AUC results, two binding site models were used to analyze protein-protein interactions. The binding isotherms were fit to determine apparent molar reaction enthalpy (Δ*H*), apparent entropy (Δ*S*), dissociation constant (*K*_D_) and stoichiometry of binding (*N*). To calculate ligand heat of dilution, experiments were performed under the same experimental conditions, except the protein in the sample cell was replaced with the buffer. In all the calculations, the ligand heat of dilution was subtracted from the data. The data was analyzed using Origin 6.0 software suite.

### Bio-Layer Interferometry (BLI)

To study the interactions of YeeF-CT^(M)^ with DNA, ForteBio Octet RED 96 (Forte Bio, Fremont, CA, United States) and streptavidin-coated (SA) sensors (Forte Bio, Fremont, CA, United States) were used. The experiment was conducted at 25°C in 20 mM HEPES pH 7.5, 150 mM NaCl as assay buffer, 30 mer 5′ biotinylated DNA was synthesized (5′-AGC ACAATTTAACACTTTTGTCAAGCGGCC-3′) and annealed with complementary DNA in the annealing buffer (20 mM Tris pH 8.0, 50 mM NaCl and 5 mM EDTA). Before use, the SA sensor tips were hydrated in assay buffer for 15 min. The wells in 96-microwell plate were filled with 200 μL of either buffer or sample in each case and agitated at 1000 rpm. 0.6 nm biotinylated double-stranded DNA was immobilized on the hydrated sensor tips (working and reference). Another reference sensor without biotinylated double stranded DNA subjected to the same procedure for double referencing to subtract non-specific protein binding with streptavidin-coated sensors. Following immobilization with biotinylated DNA, binding interaction with different concentrations (0.25–2 μM) of YeeF-CT^(M)^ was carried out, which included baseline (120 s), association (300 s), dissociation (600 s), regeneration with 10 mM NaOH and (30 s) baseline (120 s). To measure the binding kinetics of YeeF-CT^(M)^ with DNA, the sensor tip immobilized with DNA was first moved to the well, containing assay buffer with YeeF-CT^(M)^, to study the association and then moved to well, having only assay buffer to study dissociation. K_D_ was calculated using the curve fit (1:1) model using ForteBio Data Analysis 9.0 software.

### Electrophoretic Mobility Shift Assay (EMSA)

For *in vitro* DNA binding analysis, electrophoretic mobility shift assays were performed. Varying concentrations of YeeF-CT^(M)^ and YeeF-CT^(M)^/YezG (12.5–50 μM) were mixed with 50 and 80 ng of dsDNA (PCR amplified product) in buffer (20 mM HEPES pH 7.5, 150 mM NaCl, 5 mM EDTA) followed by incubation at room temperature for 30 min. The samples were resolved on 1% agarose gel, stained with ethidium bromide and visualized using gel documentation system (Syngene, United States).

## Results

### The Expression of YeeF-CT Is Toxic for Bacterial Growth and YezG Co-expression Can Neutralize the Toxic Effect

**FIGURE 2:**
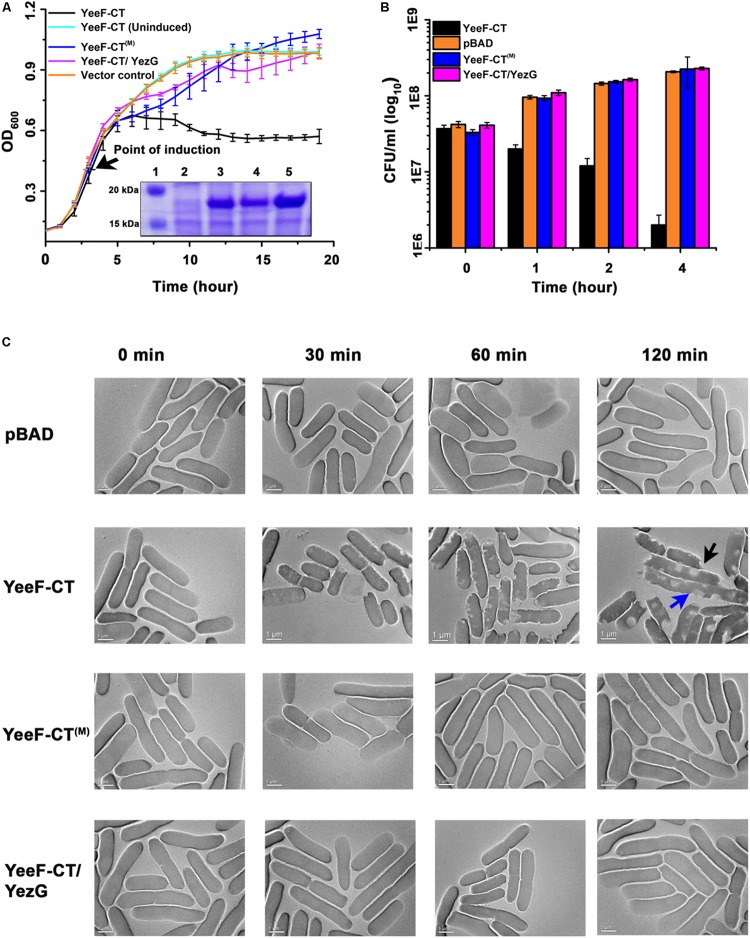
Effect of YeeF-CT expression on bacterial growth and morphology. **(A)** The growth curve assay was performed in cells expressing YeeF-CT, YeeF-CT^(M)^ and YeeF-CT/YezG and negative control. Cells expressing YeeF-CT shows reduction in OD_600_ whereas YeeF-CT^(M)^, YeeF-CT/YezG, and negative control expression did not affect cell growth. The inset shows SDS-PAGE analysis showing the expression of proteins; (1) Protein ladder; (2) Negative control; (3) YeeF-CT; (4) YeeF-CT^(M)^ and; (5) YeeF-CT/YezG. Since the molecular weight of YeeF-CT and YezG are apparently similar i.e., 18.9 and 19.2 kDa, respectively, so they appeared as a single band. **(B)** The CFU counting analysis also correlates well with the growth curve analysis. **(C)** TEM imaging of cells harboring plasmid only (upper panel), expressing YeeF-CT (second panel), YeeF-CT^(M)^ (third panel) and YeeF-CT/YezG (lower panel). While cells imaged at 0 min show smooth surface in all the samples, 30 min post-induction, cells expressing YeeF-CT appears to have some defects on the surface, which becomes quite evident at the 60 and 120 min post-induction. The blue arrow shows cell elongation and black arrow shows a defect in the cell surface. The cells expressing YeeF-CT^(M)^ and YeeF-CT/YezG appeared to have a smooth surface and intact cell wall at all the time points.

To functionally characterize YeeF-CT toxin, we transformed pBADMyc-His A-Modi-*yeeF-CT* plasmid into *E. coli* BL21(DE3) competent cells. The extracellular export of polymorphic toxins is mediated by two component secretion systems in Gram-negative bacteria and by type VII secretion system in Gram-positive bacteria. The N-terminal domain in CDI and contact dependent antagonism toxins plays a crucial role in toxin secretion (Aoki et al., 2005; Whitney et al., 2017). However, to test toxicity of YeeF-CT in the heterologous host, *E. coli*, the N-terminal domain was not included in the construct. The cognate immunity proteins on the other hand are cytosolic proteins as their primary role is to protect host cells from autoinhibition (Aoki et al., 2005; Whitney et al., 2017). So, we used native full length YezG for co-expression and neutralization studies. The expression of YeeF-CT was induced with 0.1% L-arabinose. The growth curve results show that induction of the YeeF-CT inhibits the growth of *E. coli* when compared to both uninduced cells and cells carrying empty vector (negative control) (Figure 2A). The protein expression was confirmed by SDS-PAGE analysis (Figure 2A, inset). To investigate the protective role of YezG, we co-transformed pET28a-*yezG* and pBADMyc-His A_Modi-*yeeF-CT* for the co-expression of both YezG and YeeF-CT, respectively. Cells co-expressing YeeF-CT and YezG had a similar growth curve profile as control cells (Figure 2A), indicating that co-expression of YezG protects against YeeF-CT mediated toxicity. Histidine is a catalytically important residue in several nucleases (Ho et al., 2000). Multiple sequence alignment of YeeF-CT with homologs in other bacteria suggest that His581 is highly conserved (Supplementary Figure S1). So, we mutated His581 residue to Alanine to test its potential role in the activity and constructed YeeF-CT^(M)^ point variant. The expression of YeeF-CT^(M)^ was confirmed by SDS-PAGE analysis (Figure 2A, inset). The growth curve assays show that the expression of YeeF-CT^(M)^ had no visible effect on the *E. coli* growth (Figure 2A).

Though the optical density based growth curve analysis suggests that expression of YeeF-CT causes growth arrest, we further wanted to determine the effect of YeeF-CT expression on cell viability. We performed colony forming unit (CFU) assay to study cell viability. The data suggests that the expression of YeeF-CT resulted in CFU reduction after 1-hour post-induction (Figure 2B). The CFU counts in the negative control cells, cells expressing YeeF-CT^(M)^, and cells co-expressing YeeF-CT and YezG showed no reduction in cell viability (Figure 2B). The reduction in CFU counts suggested that YeeF-CT expression is bactericidal (Figure 2B). We did not notice any observable changes in the growth curves in the initial hours post-induction, but there was a reduction in the CFU counts in the cells expressing YeeF-CT. OD based measurements do not differentiate live *vs* dead cells hence, CFU based experiments were crucial to examine the effect of toxin expression. Further, we were interested in investigating the effect of YeeF-CT expression on cell morphology. Therefore, we used transmission electron microscopy (TEM) to visualize the effect of YeeF-CT expression on the cellular morphology. The TEM imaging of cells expressing YeeF-CT, YeeF-CT^(M)^, YeeF-CT/YezG and cells harboring negative control was performed at four-time points i.e., 0, 30, 60, and 120 min post-induction (Figure 2C). At initial time points, all the cells showed normal rod-shaped morphology with intact cell membrane. The toxic effect of YeeF-CT resulted in gross morphological defects as indicated by irregular cell surfaces, membrane disruption and, cell elongation (Figure 2C). Interestingly, the cells expressing YeeF-CT^(M)^, YeeF-CT/YezG and carrying vector only show intact cells with a typical rod-shaped morphology at all the time points tested in the current study. With the increase in time, there are pronounced differences in the morphological features of the cells expressing YeeF-CT as compared to the cells expressing YeeF-CT^(M)^ and YeeF-CT/YezG toxin/immunity pair. Together these results confirmed that YeeF-CT expression was toxic to the cells and induced observable morphological changes. Also, YeeF-CT and YezG constitute a functional toxin/immunity pair, thereby demonstrating the potential role of YezG in protecting inhibitor cells from autoinhibition.

### YeeF-CT Is a Non-specific DNase and Cleaves DNA in Both *in vitro* and *in vivo* Conditions

**FIGURE 3:**
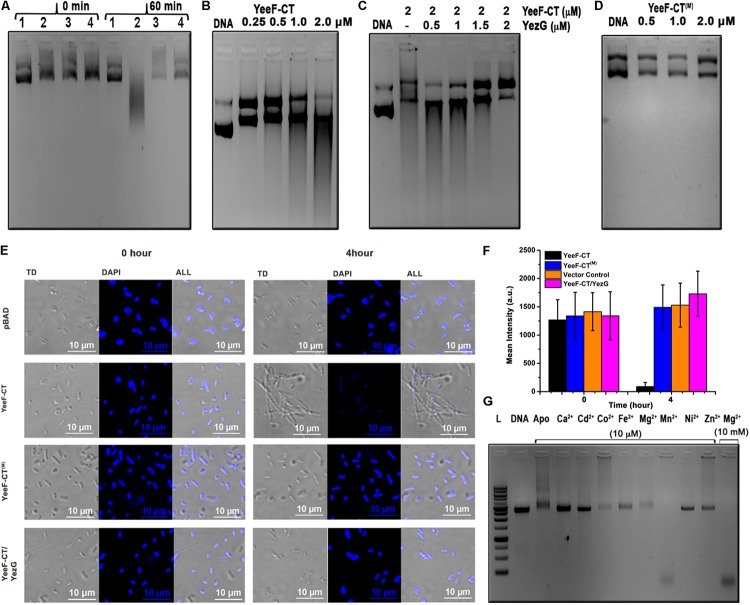
*In vitro* and *in vivo* characterization of the molecular target of YeeF-CT. **(A)** Electrophoresis of genomic DNA isolated from the cells carrying plasmid only, expressing YeeF-CT, YeeF-CT^(M)^, and YeeF-CT/YezG at 0 and 60 min post-induction. In panel **A**; 1, 2, 3, 4 represents cells carrying plasmid only, YeeF-CT expressing cells, YeeF-CT^(M)^ expressing cells, YeeF-CT/YezG expressing cells, respectively. **(B)** The agarose gel electrophoresis showing the degradation of plasmid DNA with different concentrations of purified YeeF-CT. **(C)** The agarose gel electrophoresis showing the protective role of YezG when plasmid DNA is incubated with a fixed concentration of YeeF-CT and increasing concentration of YezG. **(D)** Incubation of YeeF-CT^(M)^ with plasmid DNA showing loss of DNase activity. **(E)** The confocal microscopic images of DAPI stained *E. coli* cells expressing YeeF-CT, YeeF-CT^(M)^ and YeeF-CT/YezG showing damage to the DNA 4-hour post-induction of YeeF-CT with 0.1% L-Arabinose. **(F)** The histograms represent mean fluorescence intensity at 0 and 4 h post-induction of YeeF-CT, YeeF-CT^(M)^, YeeF-CT and YezG and negative control. **(G)** The agarose gel electrophoresis of plasmid DNA incubated with a fixed concentration of YeeF-CT and a fixed concentration of different metal ions.

To understand the molecular basis of toxicity caused by YeeF-CT in the target cells, we attempted to identify the molecular target(s) of YeeF-CT toxin. YeeF-CT is a predicted non-specific DNA/RNA endonuclease, so we investigated DNase and RNase activities. To identify the molecular target, we isolated the genomic DNA (gDNA) and total RNA from the cells expressing YeeF-CT. We could clearly observe DNA smearing in the cells over-expressing YeeF-CT while the gDNA profiles of samples before induction and negative control were similar (Figure 3A). This data suggests that YeeF-CT cleaves DNA (Figure 3A). There was no observable change in the total RNA extracted UREA-PAGE profiles of all the samples suggesting YeeF-CT does not cleave RNA (Supplementary Figure S2). The gDNA profile of the samples expressing both YeeF-CT and YezG shows gDNA profile similar to the negative control suggesting the protective role of YezG in YeeF-CT mediated toxicity (Figure 3A). To investigate whether the DNA cleavage in YeeF-CT induced toxicity is a consequence of the direct activity of YeeF-CT, we performed *in vitro* DNA cleavage assays using supercoiled plasmid DNA as substrates. YeeF-CT was able to degrade the supercoiled plasmid in a concentration-dependent manner (Figure 3B). To investigate the protective role of YezG *in vitro*, we first incubated YeeF-CT with increasing concentrations of YezG at room temperature for 45 min and then mixed this preformed complex with DNA. The results show that YezG was able to protect DNA degradation in a concentration-dependent manner (Figure 3C) further suggesting the role of YezG immunity protein as a direct inhibitor of YeeF-CT’s DNase activity. The purified YeeF-CT^(M)^ did not show the digestion of plasmid DNA (Figure 3D).

We performed confocal microscopy experiments to further probe the *in vivo* DNase activity of YeeF-CT. Cells were stained using DAPI staining. Cells transformed with pBADMyc-His A_Modi (negative control), pBADMyc-His A_Modi-*yeeF*-CT (active toxin), pBADMyc-His A_Modi-*yeeF*-CT^(M)^ (inactive toxin), pBADMyc-His A_Modi-*yeeF*-CT/pET28a-*yezG* (co-expression of toxin and immunity protein) were induced by adding appropriate inducer. Negative control cells, cells expressing YeeF-CT^(M)^, and YeeF-CT/YezG exhibited uniform DNA staining indicating intact DNA before induction and 4-hour post-induction, while fluorescence signal was highly reduced in cells expressing active toxin suggesting extensive degradation of DNA (Figure 3E). The quantitative analysis of fluorescence signals also suggests a reduction in the fluorescence signals obtained from the cells expressing YeeF-CT (Figure 3F). These results indicate that the expression of YeeF-CT causes toxicity by degrading gDNA of the cells, which could be rescued by the expression of YezG. Also, these studies further confirm the role of highly conserved His581 residue in the DNase activity of YeeF-CT.

### YeeF-CT Is a Metal-Dependent DNase

To investigate whether YeeF-CT is a metal-dependent DNase, we prepared apo-YeeF-CT by chelating metal ions from purified protein with 10 mM EDTA. The result suggests that apo-YeeF-CT does not show any DNase activity (Figure 3G) indicating that YeeF-CT is a metal-dependent DNase. However, there was upward smearing probably due to the formation of YeeF-CT: DNA complex formation. To identify the metal ion(s) responsible for DNase activity, we incubated apo-YeeF-CT with 10 μM concentration of different alkaline, earth and transition divalent metal ions. The agarose gel electrophoresis shows that depending on the metal ion supplemented, a varying degree of DNA cleavage was observed. YeeF-CT displayed robust DNA cleavage with Mn^2+^ at 10 μM physiological concentration (Anjem et al., 2009; Kaur et al., 2014). The intracellular concentration of Mg^2+^is in mM range (Cayley et al., 1991), so we also tested the activity in the presence of 10 mM Mg^2+^, the catalytic activity was equivalent to that observed for Mn^2+^. For further biochemical characterization of YeeF-CT, we investigated its DNase activity at a different time, a different temperature, a different pH level, and different salt concentrations using purified plasmid as a substrate. Our data suggest that YeeF-CT shows time-dependent cleavage of DNA substrate (Supplementary Figure S3A). The enzyme showed activity at a wide temperature range (4 to 55°C tested in this study), with the highest activity at 37°C. At 4°C we could observe a population of the relaxed nicked DNA suggesting a nickase activity (Supplementary Figure S3B). The enzyme is active in the basic pH range and up to 250 mM NaCl concentration (Supplementary Figures S3C,D).

### YeeF-CT Exists as a Homodimer in Solution and Interacts With Monomeric YezG With Nanomolar Range Dissociation Constant

**FIGURE 4:**
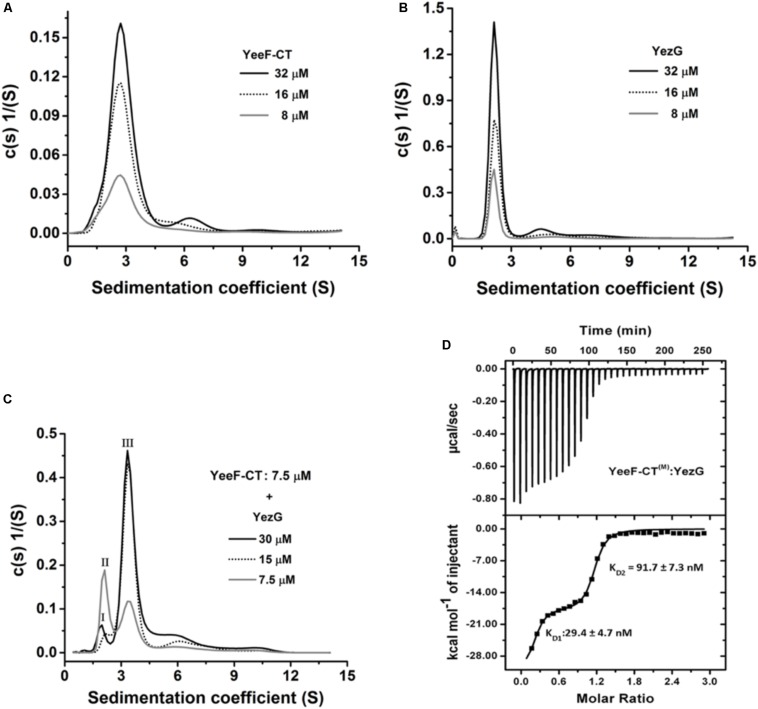
Oligomeric state and binding kinetics of YeeF-CT, YezG, and their interactions. Sedimentation coefficient distribution (s) of YeeF-CT, YezG, and YeeF- CT/YezG are shown in **panels A–C**, respectively. **(A,B)** YeeF-CT and YezG exist predominantly as a homodimer and monomer in solution, respectively. **(C)** YeeF-CT/YezG associate to form a heterotetramer in solution. Peak I and II correspond to excess YezG and YeeF-CT, respectively. Peak III corresponds to YeeF-CT/YezG complex. In all the protein samples, a minor concentration-dependent higher order oligomer population is also observed. **(D)** A representative isotherm profile of YeeF-CT and YezG interactions. The upper panel represents the calorimetric titration and the lower panel represents derived binding isotherm plotted versus the molar ratio of the YezG.

The oligomeric state plays an important role in the function of a protein. We used analytical ultracentrifugation (AUC) to examine the oligomeric states and binding stoichiometry of the YeeF-CT and YezG. AUC experiments were performed at three different concentrations of both the purified proteins at 25°C. Sedimentation velocity analysis of the purified YeeF-CT and YezG revealed a major population of 2.6 and 2.1 S, respectively. Suggesting YeeF-CT is a homodimer across the range of concentrations tested (Figure 4A) and YezG sediments as a monomer (Figure 4B). Additionally, concentration-dependent higher-order oligomers formation was observed as a minor population (∼3–5%) in both the proteins. To determine the binding stoichiometry of the complex, the concentration of YeeF-CT was fixed and titrated with increasing concentrations of YezG. A single peak corresponding to 3.4 S was observed when 7.5 μM of YeeF-CT (dimer concentration) was mixed with 15 μM of YezG (monomer concentration) indicating that a dimeric toxin molecule binds with two monomeric immunity protein molecules. (Figure 4C). Detailed parameters derived from the AUC experiments are listed in Supplementary Table S2. To further characterize YeeF-CT and YezG interactions, we used isothermal titration calorimetry (ITC). Since YeeF-CT is a homodimer and YezG exists as a monomer, we used 2-site binding to fit ITC data. The binding isotherm showed a clear biphasic binding profile suggesting cooperativity in the protein-protein interactions. The ITC data suggest the two binding sites in YeeF-CT have different affinities K_D__1_ = 29.4 ± 4.7 and K_D__2_ = 91.7 ± 7.3 nM for YezG (Figure 4D). Since YeeF-CT is a homodimer, hence there are two potential YezG binding sites and the binding of the first YezG molecule allosterically affects the binding of the other molecule. All the thermodynamic parameters are summarized in Supplementary Table S3.

### YeeF-CT/YezG/DNA Forms a Stable Ternary Complex: YezG Is an Exosite Inhibitor of YeeF-CT

**FIGURE 5:**
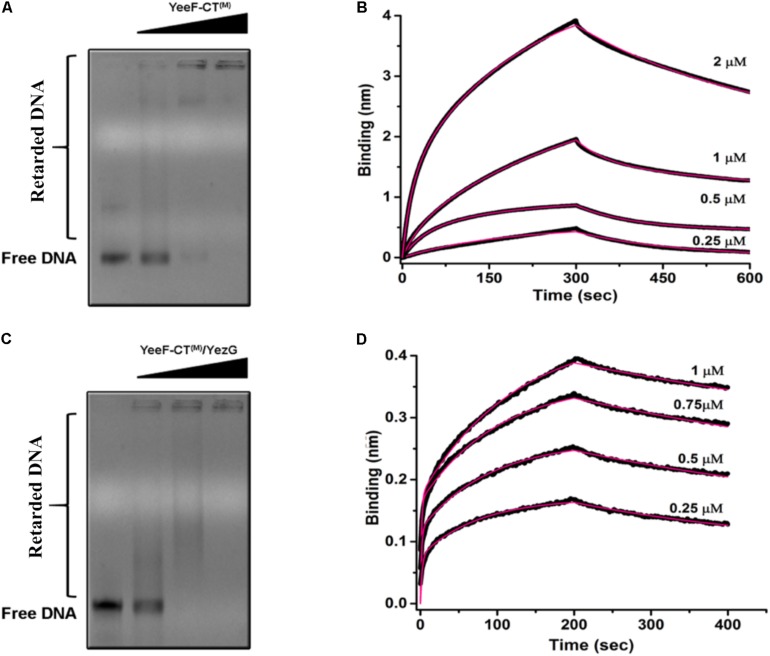
DNA binding property of YeeF-CT^(M)^ and YeeF-CT^(M)^/YezG. **(A)** YeeF-CT^(M)^ exhibits non-specific DNA binding property. A smear of retarded DNA can be visualized in the agarose gel. With an increase in the concentration of YeeF-CT^(M)^ the mobility of the DNA-protein complex is retarded and is stuck in the well. **(B)** BLI kinetics showing binding of non-specific dsDNA with YeeF-CT^(M)^. **(C,D)** Binding of YezG did not alter the DNA binding property of YeeF-CT^(M)^. **(C)** The agarose gel electrophoresis of DNA incubated with different concentrations of YeeF-CT^(M)^/YezG show retardation in DNA mobility. **(D)** BLI kinetics showing binding of non-specific dsDNA with YeeF-CT^(M)^/YezG. Both raw sensorgrams (black lines) and fitted curves (red lines) are shown in the figure.

Using YeeF-CT^(M)^active site mutant of YeeF-CT, we performed electrophoretic mobility shift assays to investigate its DNA binding property. We observed retardation in the mobility of DNA caused by binding of YeeF-CT^(M)^ in a concentration-dependent manner (Figure 5A). With the increase in the concentration of YeeF-CT^(M)^, the DNA/protein complex was unable to migrate from the wells. We further validated the DNA binding property of YeeF-CT using BioLayer Interferometry (BLI) experiments. The DNA was immobilized on the SA sensor chip. Shifts in the sensorgrams were observed during the association phase with YeeF-CT^(M)^ (Figure 5B). We also investigated the DNA binding property of YeeF-CT^(M)^ in the presence of YezG to see whether the binding of YezG inhibits substrate binding. Interestingly, the preformed complex of YeeF-CT/YezG retained the ability to bind DNA, suggesting DNA binding site on YeeF-CT did not mask the binding of YezG (Figure 5C). Upon adding the different concentrations of preformed YeeF-CT/YezG complex, we could observe a shift in the sensorgrams suggesting complex retains the ability to bind DNA (Figure 5D). YeeF-CT is a non-specific DNase so the stoichiometry of YeeF-CT^(M)^: DNA fragment used in the study is difficult to interpret as per the performed experiments. The BLI results are in good agreement with EMSA studies. Together, these results indicate that YeeF-CT retains DNA binding property in both free and YezG bound states. In other words, YezG and/or DNA binds on non-overlapping sites of YeeF-CT. Thus, YezG acts as an exosite inhibitor of YeeF-CT activity.

## Discussion

Microbes are always under constant threat from neighboring microbial competitors for limited resources and space (Hibbing et al., 2010). A recent report has demonstrated that members of the LXG family of polymorphic toxins are involved in the contact-dependent growth antagonism which may play an important role in interbacterial competition (Whitney et al., 2017). In the present study, we performed a detailed investigation on YeeF-CT/YezG, a member of the LXG family of polymorphic toxins, from *B. subtilis subsp. spizizenii str. W23*. Our results suggest that the heterologous expression of the YeeF-CT inhibits cell growth which can be blocked by the expression of YezG, a cognate immunity protein. The cytotoxicity of YeeF-CT can be attributed to its non-specific DNase activity targeting chromosomal DNA, probably beyond repair by DNA repair machinery. Although polymorphic toxins are predicted to target a diverse range of molecular targets (Zhang et al., 2011, 2012; Holberger et al., 2012), most of the experimentally characterized polymorphic toxins have nuclease activity targeting either DNA or RNA (Aoki et al., 2010; Diner et al., 2012; Morse et al., 2012; Jamet and Nassif, 2015; Johnson et al., 2016b; Ogier et al., 2016). The polymorphic toxins belong to different super-families like CdiA-CT^ECL^ toxin from *Enterobacter cloacae* ATCC 13047 and CdiA-CT^EC536^ from uropathogenic *E. coli* 536 are members of Ntox21 and Ntox28 super families, respectively (Johnson et al., 2016a, b). These toxins differ in substrate specificity, and most of them are Mg^2+^ and/or Zn^2+^ dependent nucleases (Morse et al., 2012; Johnson et al., 2016a, b) with an exception of CdiA-CT^Ykris^ from *Y. kristensenii* ATCC 33638 which is a metal independent nuclease (Batot et al., 2017). The polymorphic toxins utilize only one metal ion for their nuclease activity (Morse et al., 2012; Johnson et al., 2016a, b) with exception of CdiA-CT^EC869^, which utilizes Mg^2+^ for nickase activity and Zn^2+^ for DNase activity (Morse et al., 2012). In the previous study, members of the PF04740 family revealed RNase activity in YobL-CT, YxiD-CT and YqcG-CT domains from *B. subtilis* 168 (Holberger et al., 2012). However, YeeF-CT from *B. subtilis* 168 was not functionally characterized. In this study, we show that YeeF-CT is a metal-dependent DNase and requires Mg^2+^or Mn^2+^ ion(s) for its activity. Further biochemical characterization of YeeF-CT suggests that this enzyme is active in diverse conditions like elevated temperature, alkaline pH and low salt conditions. So, the data presented here add DNA to the list of the molecular targets of PF04740 family toxins and hence this family of toxins can be broadly classified as nucleases having either RNase or DNase activities.

In this study, we demonstrate that DNA/YeeF-CT/YezG can form a ternary complex indicating substrate binding site and the immunity protein binding sites are spatially quite distinct. This kind of immunity proteins are termed as “exosite” inhibitors of enzymes (Kleanthous and Walker, 2001). Exosite is the secondary binding site distant from the active site present in many enzymes (Kleanthous and Walker, 2001). This mode of inhibition is also present in CdiA-CT_*o*__11_^EC869^/CdiI toxin immunity complex from *E. coli* O157:H7 (Morse et al., 2012) and colicin (E3, 7, 8, 9) where they utilize the exosite and neutralize the toxins indirectly by distorting the active site and preventing binding of the substrate (Ko et al., 1999; Mosbahi et al., 2004; Lancaster et al., 2008; Meenan et al., 2010). However, there are no reports where the toxin/immunity complex also interacts with the DNA substrate. The spatial exclusion of immunity protein and substrate binding sites probably provides new opportunities to evolve novel protein-protein interaction interfaces while maintaining the integrity of the active site and thus toxic activity. This provides a selective advantage over the neighboring bacteria having closely related toxins.

Using AUC experiments, we are able to unambiguously assign oligomeric states of YeeF-CT and YezG and binding stoichiometry of the complex. To the best of our knowledge, this is the first polymorphic toxin which exists as a homodimer in solution. It is still unclear whether dimerization is required for active site formation at the interface, or enhances stability, or is involved in some other function. Future structural studies may help us in understanding this better. The oligomeric state of YeeF-CT and YeeF-CT/YezG is unique among structurally characterized toxin/immunity pairs from other PTS (Morse et al., 2012, 2015; Johnson et al., 2016a, b). To further investigate the thermodynamic parameters of this interaction, we used ITC. Nanomolar range affinity of YeeF-CT/YezG is comparable to other PTS, CdiA-CT^536^/CdiI^536^ (K_D_ = 26.4 ± 2.1 nM) (Kaundal et al., 2016), CdiA-CT_II_^Bp1026b^/CdiI_II_^Bp1026b^ (K_D_ = 21.1 ± 9 nM) and CdiA-_CTO__11_^EC869^/CdiI^EC869^ (K_D_ = 17.8 ± 7 nM) (Morse et al., 2012) studies on Gram-negative bacteria. The ITC data suggests an allosteric mode of binding as two sites on YeeF-CT dimer have ∼3.5-fold difference in affinity for YezG molecules. This suggests that the binding of one YezG molecule may probably lead to consequent conformational rearrangements, allosterically reducing the affinity for the second molecule. To summarize, we have discovered a metal ion(s) dependent bacterial DNase. Our data also suggests that YezG is an exosite inhibitor of YeeF-CT, where the preformed toxin/immunity complex retains the ability to bind to substrate DNA (Figure 5). Our lab is currently working on solving the high-resolution structures of YeeF-CT/YezG, YeeF-CT/YezG/DNA and YeeF-CT/DNA complexes to better understand the molecular basis for DNA binding and unravel the allosteric mechanism involved in toxin/immunity protein interactions.

## Data Availability Statement

The datasets generated for this study are available on request to the corresponding author.

## Author Contributions

KT conceived the idea. KT, SK, and AD planned the experiments, analyzed the data and wrote the manuscript. SK, AD, and GK performed the experiments.

## Conflict of Interest

The authors declare that the research was conducted in the absence of any commercial or financial relationships that could be construed as a potential conflict of interest.
