# Supplementary material for: Molecular and Biochemical Characterization of YeeF/YezG, a Polymorphic Toxin-Immunity Protein Pair From Bacillus subtilis
Source: Front Microbiol. 2020 Feb 14;11:95. doi: 10.3389/fmicb.2020.00095 (PMC7033585; doi:10.3389/fmicb.2020.00095)

Supplementary Information

Molecular and biochemical characterization of YeeF/YezG, a polymorphic toxin-immunity protein pair from *Bacillus subtilis*

Running title: *YeeF/YezG*, a polymorphic toxin-immunity system.

Soni Kaundal[1,2], Amar Deep[1], Gundeep Kaur[1,3], and Krishan Gopal Thakur[1]*

[1]Structural Biology Laboratory, G. N. Ramachandran Protein Centre, Council of Scientific and Industrial Research-Institute of Microbial Technology (CSIR-IMTECH), Chandigarh-160036, INDIA.

[2] **Current address:** Verna and Marrs McLean Department of Biochemistry and Molecular Biology Baylor College of Medicine, One Baylor Plaza, Houston, TX 77030.

[3]**Current Address:** Department of Epigenetics and Molecular Carcinogenesis, University of Texas, MD Anderson Cancer Centre Houston, Texas 77030, USA.

* To whom correspondence should be addressed.
Email: krishang@imtech.res.in
Tel: +911726665470; Fax: +91-172-2690585;

Keywords: Contact-dependent antagonism, Toxin-immunity system, Polymorphic toxin system, Bacterial toxin, Protein-protein interactions.

Table S1: Table showing the cloning of YeeF-CT$_{(M)}$, YezG and YeeF-CT$_{(M)}$/ YezG

**Gene**	**Size (bp)**	**Vector**	**Cloning sites**
***yeeF*-CT**	426	pBAD/Myc-His A_Modi	NheI/HindIII
***yeeF*-CT$_{(M)}$**	426	pBAD/Myc-His A_Modi	NheI/HindIII
yezG	486	pET28a	NdeI/XhoI

Table S2: Table summarizing the parameters obtained from AUC of YeeF-CT$_{(M)}$, YezG and YeeF-CT$_{(M)}$/ YezG (where MW_{cal} and MW_{obs}; calculated and observed Molecular weights of the purified protein and the protein-protein complex, O.S; Oligomeric state/ Stoichiometry).

**Sample**	**$MW_{cal(kDa)}$**	**$MW_{obs(kDa)}$**	**O.S**	**S_w(S)**	**Frictional ratio**
**YeeF-CT$_{(M)}$**	18.3	35.2	Dimer	2.6	1.55
**YezG**	19.2	19.7	Monomer	2.1	1.4
**YeeF-CT$_{(M)}$/ YezG**	37.5	70.2	Heterotetramer	3.4	1.6

Table S3: Table summarizing the thermodynamic parameters of YeeF-CT$_{(M)}$/ YezG interactions using two site binding model.

**Protein samples**	**ITC**			
	**N**	**ΔH (cal/mol)**	**ΔS (cal/mol/K)**	**K_D (nM)**
YeeF-CT$_{(M)}$/ YezG	0.2<br>0.92	-3.08e4 ± 5.78e3<br>-1.78e4 ± 667	-64.4<br>-27.7	29.4 ± 4.7<br>91.7 ± 7.3

Fig. S1: Multiple sequence alignment of YeeF-CT with other bacterial homologs showing the conserved Histidine present in the active site. The black rectangular box shows the conserved histidine residue present in the active site of the nucleases.

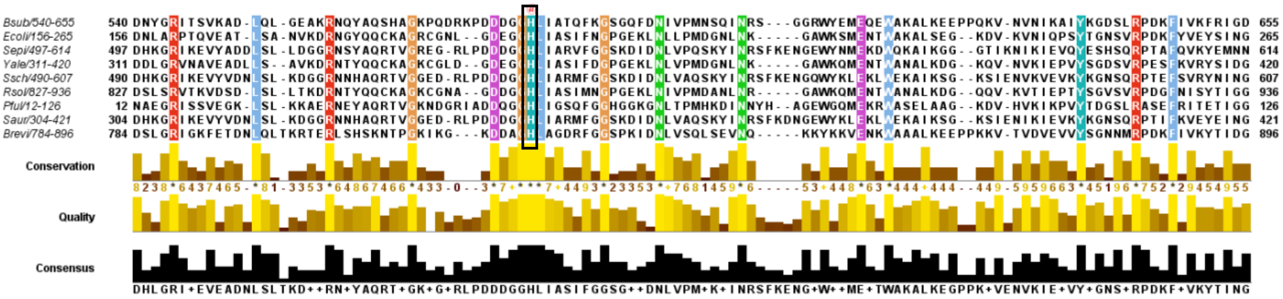

Bsub: Bacillus subtilis, Ecoli: Escherichia coli, Sepi: Staphylococcus epidermidis, Yale: Yersinia aleksiciae, Ssch: Staphylococcus schweitzeri, Rsol: Ralstonia solanacearum, Pful: Pseudomonas fulva, Saur: Staphylococcus aureus, Brevi: Brevibacillus brevis

Fig. S2: (A) UREA-PAGE profiles of total RNA extracted from cells expressing YeeF-CT and YeeF-CT$_{(M)}$, and time of induction, post 2 hour induction and post 4 hour induction. The extracted total RNA was resolved on 6% UREA-PAGE and stained with ethidium bromide dye. ssRNA Low range ladder was used as a molecular weight marker. **(B)** *In vitro* RNase activity performed using 5 μM each of purified YeeF-CT and YeeF-CT$_{(M)}$. The total RNA extracted from cells was incubated with purified proteins and incubated from 5 to 20 mins at 37 °C. No observable differences in the RNA UREA-PAGE profile were observed in the presence of both the active and inactive protein variant. However, trace RNase activity observed in the samples treated with YeeF-CT and YeeF-CT$_{(M)}$ may be due to co-purified RNases or other unknown reasons. The samples were resolved on 6% UREA-PAGE and stained with ethidium bromide dye. ssRNA ladder from NEB was used as a molecular weight marker.

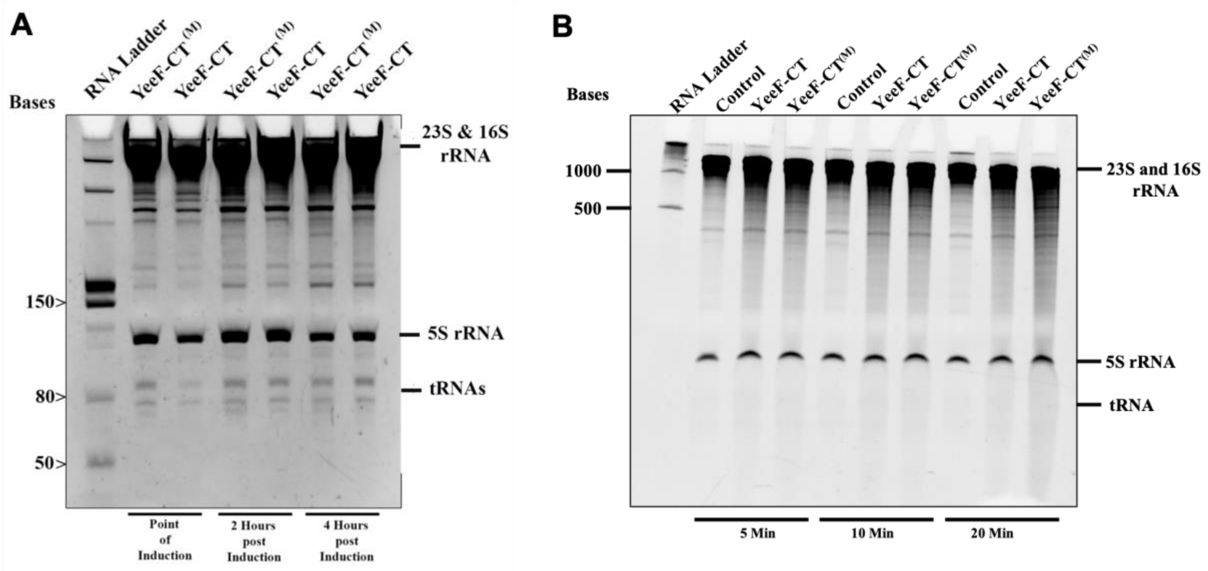

Fig. S3. Biochemical characterization of the DNase activity of YeeF-CT. Agarose gel analysis of the **c**leavage of DNA substrate at different time **(A),** temperature **(B),** pH **(C),** and salt concentrations **(D).** At low temperature the nicked relaxed, linear and uncleaved DNA can be seen clearly. The maximum cleavage was observed at 37 ˚C in this experimen**t.**

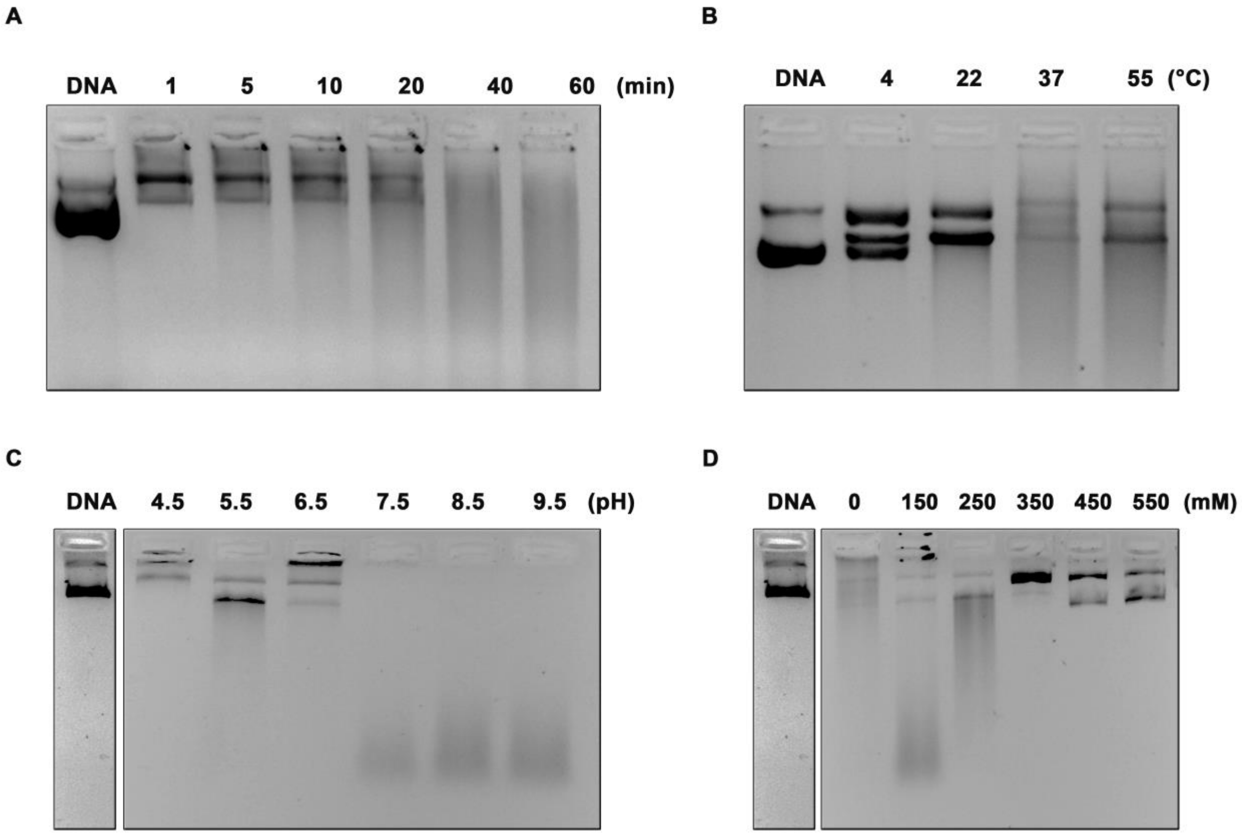

Supplement: Supplementary file 1 [file Data_Sheet_1.pdf]
